# The Pathophysiological Functions of Heparanases: From Evolution, Structural and Tissue‐Specific Perspectives

**DOI:** 10.1096/fj.202501859R

**Published:** 2025-09-03

**Authors:** Elham Vahdatahar, Clément Daviaud, Hugo Main, Rachel Havret, Claire Debarnot, Laure Favot‐Laforge, Jean‐François Jégou, Ingrid Fruitier‐Arnaudin, Antoine Dufour, Romain Vivès, Franck Morel, Yves Bourne, Kévin Baranger

**Affiliations:** ^1^ Aix‐Marseille Univ, CNRS, AFMB Marseille France; ^2^ LIENSs UMR 7266, CNRS‐La Rochelle Université La Rochelle France; ^3^ Université de Poitiers, LITEC, UR15560 Poitiers France; ^4^ Department of Physiology and Pharmacology, McCaig Institute for Bone and Joint Health Hotchkiss Brain Institute, Snyder Institute for Chronic Diseases, Cumming School of Medicine, University of Calgary Calgary Alberta Canada; ^5^ Department of Biochemistry and Molecular Biology, McCaig Institute for Bone and Joint Health Hotchkiss Brain Institute, Snyder Institute for Chronic Diseases, Cumming School of Medicine, University of Calgary Calgary Alberta Canada; ^6^ University of Grenoble Alpes, CNRS, CEA, IBS Grenoble France

**Keywords:** cancer, Heparanase, immune cell, molecular modeling, mouse model, skin inflammation

## Abstract

Heparanase 1 (HPSE1) is a unique endoglycosidase responsible for the enzymatic cleavage of heparan sulfate, thereby playing important functions in cancer processes. In contrast, the structurally related Heparanase 2 (HPSE2) lacks catalytic activity and appears to counteract HPSE1 activities. However, contradictory observations in various pathologies highlight the need for a better understanding of the respective contributions of both heparanases. In this review, we provide a comprehensive resource about the biology of HPSE1 and HPSE2 based on findings from different mouse models, with an emphasis on immune cells and their involvement in skin pathophysiology. In addition, we explore the evolutionary relationships between the two heparanases and describe the structure–function of HPSE2 using the advanced protein‐prediction tool AlphaFold 3 (AF3). These approaches unveil new insights for deciphering the functional molecular determinants that distinguish HPSE1 from HPSE2.

## Graphical abstract

Heparanase 1 (HPSE1) and Heparanase 2 (HPSE2) exhibit opposing expression patterns and functions in pathological contexts, such as cancer. Here, we describe their specific expression in immune cells and their implications in skin diseases. Although they are structurally similar and considered to be ‘cousins’, we found no common ancestor and that HPSE2 is better conserved than HPSE1. We also proposed a molecular model to explain how HPSE2c could interact with and inhibit HPSE1.

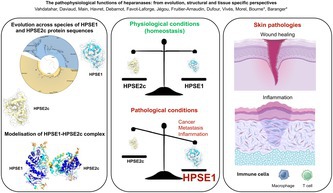

**Abbreviations**
AF3: AlphaFold 3
BM: basement membrane
Bptf: bromodomain PHD finger transcription factor
CIA: collagen‐induced rheumatoid arthritis
CTSL: Cathepsin L
DCs: dendritic cells
DEJ: dermal‐epithelial junctions
DTH: delayed‐type hypersensitivity
ECM: extracellular matrix
EGR1: Early Growth Response
ERK1/2: extracellular signal‐regulated kinase 1/2
GABP: GA‐binding protein
GAG: glycosaminoglycan
GH79: glycoside hydrolase 79
GlcNAc: N‐acetylglucosamine
GlcNS: N‐sulfoglucosamine
HDM: House Dust Mite
HexUA: hexuronic acid
HME: hereditary multiple exostoses
HPSE1/2: human Heparanase 1/2
HS: Heparan sulfate
HSPGs: Heparan sulfate proteoglycan
IdoUA: iduronic acid
IFN‐γ: interferon‐γ
IMQ: imiquimod
Jnk: c‐Jun N‐terminal kinase
Mapk: mitogen‐activated protein kinase
MBP: major basic protein
MCs: mast cells
MMPs: matrix metalloproteinases
NHEK: normal human epidermal keratinocytes
NK: natural killers
PPS: Pentosan polysulfate sodium
rmsd: Root‐Mean‐Square Deviation
SAA: serum amyloid A
Sp1 and Sp3: specificity protein 1 and 3
TPA: 12‐O‐tetradecanoyl phorbol 12‐myristate 13‐acetate
UFS: urofacial syndrome

## 1 Introduction

Heparan sulfate (HS), a member of the glycosaminoglycan (GAG) family, is composed of a linear repeating D‐HexUA(β1 → 4)D‐GlcNX(α1 → 4)]_n_ disaccharide motif, in which HexUA is either glucuronic acid (GlcUA) or its C5 epimer, iduronic acid (IdoUA) and GlcNX is either N‐acetylglucosamine (GlcNAc) or N‐sulfoglucosamine (GlcNS) [1]. Further diversity to this disaccharide backbone structure is provided by O‐sulfation, at C‐2 of IdoUA as well as C‐6 and more rarely C‐3 of GlcNX.

The structure of HS is tightly regulated during a multi‐step biosynthesis process involving enzymatic activities responsible first for the attachment to a core protein, then for the polysaccharide polymerization and modifications (epimerization and sulfation). Additionally, post‐synthetic mechanisms further regulate HS structure through the action of extracellular enzymes, including heparanase endoglycosidase and endosulfatases of the Sulf family [2, 3]. HS chains are embedded into heparan sulfate proteoglycans (HSPGs) where they are covalently linked to core proteins either at the cell surface (e.g., syndecans 1–4, glypicans 1–6, CD44v3, neuropilin‐1, betaglycan) or as secreted proteins (e.g., agrin, perlecan, collagen XVIII) [2, 4]. HSPGs are ubiquitous components of the cell surface and extracellular matrix (ECM) particularly in the basement membrane (BM) and are involved in major structural and signaling functions including physiological, homeostatic and pathological processes such as embryogenesis, morphogenesis, development, inflammation, angiogenesis, cancer metastasis and several others. These activities, i.e., structural and/or signaling functions, are typically mediated through interactions of HS with a wide array of proteins [2, 4].

## 2 Heparanase 1

Heparanase 1 (HPSE1 thereafter), an endo‐β‐D‐glucuronidase of the glycoside hydrolase 79 (GH79) family in the CAZy database (https://www.cazy.org/GH79.html, [5]), is the sole enzyme responsible for HS breakdown and cleaves HS chains at GlcUA residues within specific motifs as described by Rivara et al. [6]. In late endosomes and lysosomes, HPSE1 plays an essential housekeeping role in the catabolic processing of internalized HSPGs. However, it can also be trafficked to the cell surface and released into the ECM, where it degrades extracellular pools of HS chains. When expressed at low levels in most tissues, if not all cells, HPSE1 contributes to the physiological maintenance of ECM [3], whereas abnormal HPSE1 overexpression potently drives the growth of aggressive metastatic cancers [7, 8, 9, 10]. Through both experimental overexpression and gene silencing approaches, HPSE1 has been implicated in cell invasion, metastasis, and angiogenesis [11, 12, 13] and its role in inflammatory processes has also been demonstrated [7, 9, 14]. It must be noted that the non‐enzymatic functions of HPSE1 also contribute to cancer. For example, a splice variant of HPSE1 named T5, devoid of enzymatic activity, exhibits pro‐tumorigenic properties [15]. Several studies have revealed an inverse relationship between *HPSE1* expression levels and the methylation status of its CpG promoter region. Indeed, hypermethylation of its promoter region has been associated with lower expression in different cancer cell lines such as breast cancer cells (MCF‐7), human choriocarcinoma cells (JAR) or rat glioma cells (C‐6). Suppression of cytosine methylation using the demethylating agent (5‐azacytidine) resulted in increased HPSE1 mRNA and protein expression, as well as enhanced enzymatic activity [16, 17]. Several transcription factors have been described to promote *HPSE1* gene expression. Interestingly, simultaneous hypomethylation of its promoter, particularly at Early Growth Response 1 (EGR1) binding sites, combined with EGR1 upregulation, led to increased *HPSE1* expression in prostate cancer [18]. While EGR1 seems to upregulate HPSE1 in tumor cells and T lymphocytes via an ERK1/2 (extracellular signal‐regulated kinase 1/2) dependent pathway [18, 19, 20, 21, 22], it exerts the opposite effect in human and mouse melanoma cells [19]. Other transcription factors, such as ETS1 and ETS2, GA‐binding protein (GABP) and specificity protein 1 and 3 (Sp1 and Sp3) directly contribute to the upregulation of *HPSE1* expression. GABP and Sp1 not only individually induce *HPSE1* expression, but also interact synergistically to enhance its expression [23, 24]. Additionally, the wild‐type p53 protein, a central regulator of the cell cycle and apoptosis [25], actively represses *HPSE1* expression by directly binding to its promoter under homeostatic conditions. However, oncogenic mutations in p53 lead to the dysregulation of HPSE1 levels by removing its repressor effect [26]. Furthermore, the transcription factor NF‐κB, known for its pivotal role in various pathological processes including inflammation and cancer, also acts as a strong activator of *HPSE1* gene expression in tumor cells [8, 27, 28].

HPSE1 actively contributes to diverse roles during inflammatory processes, including immune cell infiltration and macrophages activation [29]. Among other HPSE1 regulators, several pro‐inflammatory cytokines, such as interferon‐γ (IFN‐γ), tumor necrosis factor‐α (TNF‐α), monocyte chemoattractant protein‐1 (MCP‐1) and interleukin (IL)‐1β, IL‐2, IL‐15, IL‐17, upregulated *HPSE1* gene expression [30, 31, 32, 33, 34, 35, 36]. Altogether, this might explain the dysregulation of HPSE1 observed in cancer and inflammatory diseases.

**FIGURE 1:**
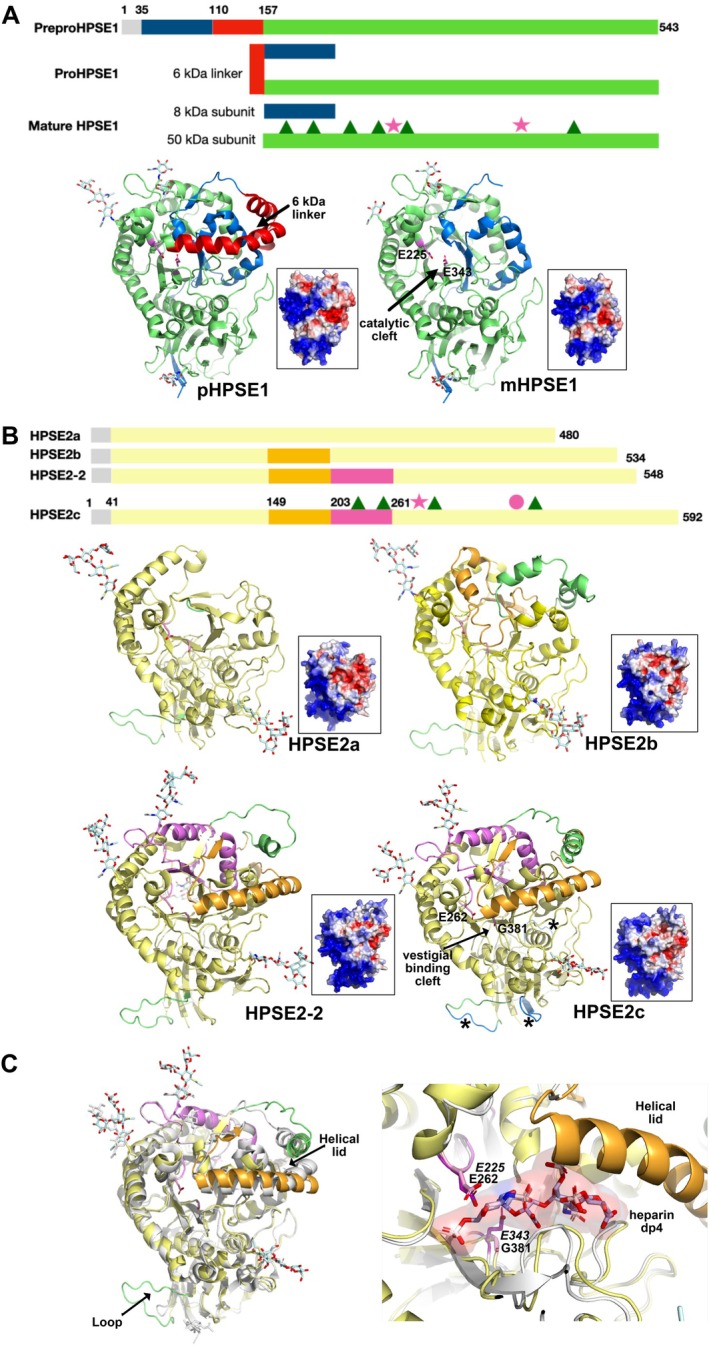
(A) (Top) Schematic view of the HPSE1 architecture and maturation steps (prepro to the mature form from top to bottom) with the small (8 kDa), the linker (6 kDa) and large (50 kDa) subunit colored in blue, red and green, respectively. Triangles and stars denote the position of the N‐glycosylation sites and catalytic residues (Glu225 and Glu343), respectively. (Bottom) Overall views of the proHPSE1 (accession code 5la4) and mature HPSE1 (accession code 5e8m) crystal structures with the subunits colored as in A and viewed in a similar orientation looking down the active site cleft; the distribution of the electrostatic potential mapped on the molecular surface at −3 kT/e (red) to +3 kT/e (blue) is shown as inset. (B) (Top) Schematic architecture of the four human HPSE2 isoforms (HPSE2a, HPSE2b, HPSE2‐2 and HPSE2c from top to bottom) with the corresponding AF3 predicted structures viewed in a similar orientation and looking down the vestigial binding cleft, mapped by the electrostatic potential as inset. The position of the predicted N‐glycosylation sites (triangles colored in green for those conserved with HPSE1) and the vestigial residue pair (Glu262 and Gly381 as a pink star and circle) in HPSE2c are indicated. Differences in the overall architecture of the four HPSE2 isoforms due to the spliced segments within the (β/α)_8_ domain and in the topology of the electropositive patches between HPSE1 and HPSE2 isoforms are evident. The three putative heparin‐binding motifs in HPSE2c are colored in blue and indicated by an asterisk. (C) (Left) Overlay of HPSE2c and proHPSE1 (colored as in B) showing the two surface regions that differ in HPSE2c colored in green. (Right) Close‐up view of the HPSE1 binding cleft in the structure of HPSE1‐heparin tetrasaccharide dp4 complex (accession code 5e9c) overlay on HPSE2c showing the steric clashes of the dp4 substrate with the HPSE2c helical lid.

At the molecular level, HPSE1 is synthesized as a 65 kDa latent, catalytically inactive precursor (proHPSE1) that subsequently undergoes Cathepsin L (CTSL)‐dependent proteolytic cleavage to remove a 6 kDa linker peptide, yielding an 8 kDa and a 50 kDa subunit that heterodimerize to form the active enzyme [37] (Figure 1A). A comparative analysis of the crystal structures of the pro‐ and mature forms of HPSE1, which features a (β/α)_8_ barrel flanked by a smaller β‐sandwich domain, has evidenced the activation mechanism of HPSE1 being triggered by the helical 6 kDa linker in proHPSE1 that sits above and clutters the active site cleft, preventing access to the bulky HS substrates. In mature HPSE1, the opened cleft is lined by Glu343 and Glu225 as, respectively, the catalytic nucleophile and acid–base residues [38] (Figure 1A).

## 3 Heparanase 2

An ortholog of HPSE1 has been identified and named Heparanase 2 (HPSE2 thereafter). HPSE2 shares an overall sequence identity and similarity of ~40% and 59% with HPSE1 and is predicted to encode four protein isoforms (HPSE2a, HPSE2b, HPSE2c and HPSE2‐2) generated by alternative splicing (Figure 1B) [39, 40, 41]. Compared to the larger HPSE2c, which is more prone to being secreted likely due to its higher glycosylation level, the two smaller non‐secreted (intracellular) HPSE2a and HPSE2b isoforms lack segments of 110 and 57 residues, respectively, in the (β/α)_8_ barrel domain (Figure 1B) [39, 41]. The fourth transcript that encodes a 548‐residue‐length HPSE2‐2 isoform has a 44‐residue deletion at the C‐terminus compared to HPSE2c [40]. The three (a, b and c) HPSE2 isoforms have been detected in plasma by western blot, and their levels are higher in gastrointestinal carcinoma patients than healthy controls [42].

Unlike HPSE1 [41], HPSE2 does not seem to undergo a maturation process and lacks catalytic activity toward HS as only the Glu acid–base residue is conserved at the vestigial active site. Instead, the HPSE2c isoform exhibits high affinity toward heparin and HS and acts as an inhibitor of HPSE1, either by competing for substrates or, more surprisingly, by directly interacting and forming physical contacts with HPSE1 [43, 44]. To further explore the inhibitory mechanism of HPSE1 by HPSE2, two HPSE2c‐derived peptides that encompass residues 381–400 and 401–430 within the (β/α)_8_ barrel domain have been shown to inhibit HPSE1 activity in a dose‐dependent manner, as also observed for full‐length HPSE2c, emphasizing the formation of a physical protein complex [44].

In contrast to HPSE1, which is mainly overexpressed in pathological processes, HPSE2 is weakly expressed in many cancers, such as gastric and breast cancers [10, 45] and in turn behaves as a tumor suppressor [46, 47]. Indeed, in head and neck cancer, overexpression of *HPSE2* leads to increased SOX2 levels, reduced tumor growth and vascular density, and higher HPSE2 levels were associated with better survival of patients [10, 48, 49, 50]. This finds support in colorectal cancer studies, where HPSE2 underexpression is associated with a higher risk of cancer‐related death. The underexpression is linked to the hypermethylation of CpG islands on the *HPSE2* promoter [51]. In addition to cis‐regulation mechanisms, *HPSE2* expression also appears to be increased in response to various stresses, such as heat shock, proteotoxic stress, ER stress and lysosomal stress. This regulation is thought to depend on the binding of activating transcription factor 3 to its promoter [52]. More recently, it has been demonstrated that HPSE2 may counteract HPSE1‐mediated pathogenic processes, including inflammation [44, 53].

Altogether, HPSE1 and HPSE2 have a dichotomous relationship and seem to behave as “the bad cop and the good cop” respectively, in cancer and inflammatory processes. Herein, we review current knowledge on heparanases in immune cells and in pathophysiology in the context of experimental mouse models, with a specific focus on skin diseases. Finally, we discuss the intriguing interactions and relationships between these two “look‐alike” proteins from an evolutionary and molecular modeling point of view.

## 4 Mouse Model to Study Hpse1 and Hpse2

In 2004, a first mouse model overexpressing HPSE1, namely, Hpa‐Tg, allowed characterization of the in vivo functions of HPSE1 [54]. In this model, the chicken β‐actin promoter drove human *HPSE1* expression in all tissues. Depending on the tissues studied, a 6‐ to 30‐fold increase in HS degradation was detected in Hpa‐Tg compared to WT. These mice had no obvious physical abnormalities but consumed less food, resulting in a smaller body size than WT mice, and additionally exhibited more branched mammary glands and accelerated hair growth [54]. In 2009, the first *Hpse1* knockout (*Hpse1* ^*−/−*^) mouse model, generated by targeting exon 1 by homologous recombination, was described. These mice exhibited a normal life span without notable phenotype abnormalities but produced more homogeneous and elongated HS chains compared with WT mice [55]. Unexpectedly, they showed increased mammary branching, sprouting and angiogenesis (neovascularization). These activities were associated with higher expression levels of two matrix metalloproteinases (MMPs), *Mmp‐2* and *Mmp‐14*, where β‐catenin seemed to be driving *Mmp‐14* expression. It was hypothesized that MMPs' activities could compensate for the lack of Hpse1 on common substrates such as HSPGs [55], although this remains to be clearly demonstrated. However, such HPSE1 and MMP‐14 co‐regulation was not observed in the MDA‐MB‐231 cell line, where decreased levels of *HPSE1*, induced by treatment with small oligomers of λ‐carrageenan or by shRNA strategy, were associated with a reduction of *MMP‐14* expression and activity [56].

Interestingly, contrary to its pro‐migratory functions described in cancer cells, Hpse1 has no impact on macrophage migration and lymphocyte homing in the airway lumen [57]. While lymphocyte morphology remained unchanged compared to WT, *Hpse1* ^−/−^ alveolar macrophages, but not peritoneal, accumulated granules composed of needle‐shaped crystalline structures of Ym1, a chitinase‐like protein, and exhibited longer HS chains compared with WT mice [57]. Dendritic cell (DC) transmigration was significantly delayed in *Hpse1* ^*−/−*^ mice in vitro and in vivo, but not abolished, likely due to compensatory upregulation of *Mmp‐14* expression. Interestingly, DCs from *Hpse1* ^*−/−*^ mice demonstrated enhanced phagocytosis of apoptotic cells [58]. The interplay between Hpse1 and Mmps was suspected to be responsible for AA‐amyloidosis resolution in *Hpse1* ^*−/−*^ mice, where upregulation of Mmp‐9, along with that of Mmp‐2, might facilitate degradation of serum amyloid A (SAA), thereby reducing SAA deposition in *Hpse1* ^*−/−*^ mice [59].

HS are massively enriched in the basement membrane of inflamed skin vessels, and no differences were observed between *Hpse1* ^*−/−*^ and WT mice. This suggests that Hpse1 is not directly implicated in this phenomenon, which probably ensures an efficient chemokine gradient formation required for leukocyte recruitment [60]. Hpse1 produced by lymphocytes or neutrophils is not required for their normal homing upon skin inflammatory stimulation, whereas *Hpse1* ^−/−^ monocytes were less efficiently recruited during zymosan‐induced peritoneal inflammation [61]. In *Hpse1* ^−/−^ mice, diabetic nephropathy and experimental glomerulonephritis were reduced, with less albuminuria and renal damage. This was associated with higher HS levels, reduced glomerular macrophage and leukocyte influx and reduced levels of inflammatory cytokines [62, 63]. Vitamin D dampens *Hpse1* expression in rat podocytes and improves proteinuria in a model of nephropathy [64]. Moreover, CtsL activity also contributes to experimental diabetic nephropathy, as *Ctsl* ^−/−^ mice showed no detectable Hpse1, reduced renal macrophage infiltration and no albuminuria [65]. Mechanistically, *Hpse1* ^−/−^ macrophages displayed impaired inflammatory signaling pathways involving Erk, p38, Jnk (c‐Jun N‐terminal kinase) activation and c‐Fos levels, leading to reduced inflammatory cytokine production, independent of Hpse1 enzymatic activity. Moreover, Hpse1 is required for maintaining macrophage phagocytosis capacity, which ensures the dampening of pro‐tumorigenic effects [66]. This role of Hpse1 in tumor‐associated macrophages was demonstrated in obesity‐related cancer progression [67]. Interestingly, *Hpse*1^−/−^ mice exhibited retinal morphology defects resembling those observed in humans treated with the FDA‐approved HPSE1 inhibitor Pentosan polysulfate sodium (PPS; Elmiron) for bladder pain and interstitial cystitis [68].

Some of these findings were reassessed using another constitutive *Hpse1* ^*−/−*^ mouse model developed by Poon et al. [69] though with partly contradictory results. This model also targeted exon 1 but used a different strategy, i.e., crossing *Hpse1* ^flox/flox^ mice with TNAP‐Cre mice, to delete floxed genes in primordial germ cells by E9. These *Hpse1* ^*−/−*^ mice exhibited no obvious anatomical phenotype with normal fertility and life span as reported by Zcharia et al. [55]. However, they observed impaired DC migration compared to WT, while the response to inflammation remained normal. Unlike previous studies, Mmp–2, –9 and –14 were not upregulated, but House Dust Mite (HDM)‐induced allergic inflammation was reduced in *Hpse1* ^*−/−*^ mice compared to WT [69]. In a recent study, spontaneous mammary tumor‐developing MMTV‐PyMT mice crossed with these *Hpse1* ^*−/−*^ mice were used to study the role of Hpse1 in breast cancer [68]. Tumors developed normally in the presence or absence of Hpse1, and Mmp levels were not changed in the absence of Hpse1 as demonstrated earlier [55]. These results suggested that mammary tumor angiogenesis, progression and metastasis were Hpse1‐independent [70]. In Apolipoprotein E deficient mice fed with high fat diet, Hpse1 was shown to promote atherosclerosis onset and progression, associated with higher levels of MCP‐1 as well as leukocytes and DCs recruitment [71]. Overall, these data advocate a more comprehensive and selective targeting of HPSE1 in tumors to avoid detrimental effects on effector T cell infiltration, while also supporting HPSE1 inhibitors as potential treatments in cardiovascular disease.

HPSE1 levels are elevated in Hereditary Multiple Exostoses (HME), a rare pediatric disorder characterized by osteochondromas, caused by mutations in the *EXT1* or *EXT2* genes, encoding two HS‐synthesizing enzymes [3]. Using new *Hpse1* ^*−/−*^ mice generated by CrispR/Cas9, crossed with *Ext1/Ext2* ^*−/−*^ mice, it was shown that Hpse1 is not implicated in cancer initiation or progression [72]. Overall, these mouse models suggested contrasting activities of Hpse1, with significant implications for HPSE1‐targeted therapies in cancer and other diseases.

Regarding HPSE2, there are far less experimental data compared to HPSE1, first because it was discovered later and is devoid of enzymatic activity [41], and secondly because *Hpse2* ^−/−^ mice died within one month [73]. Interestingly, *HPSE2* mutations in humans are associated with urofacial syndrome (UFS), also known as Ochoa disease [74]. The constitutive *Hpse2* ^−/−^ mice early recapitulated this phenotype before dying, suggesting a key function for HPSE2 at neuromuscular junctions to control muscles of the face and the bladder [73]. Encouraging data have come from the generation of conditional *Hpse2* ^−/−^ mice, which are viable [75]. These mice displayed a smaller and inflamed pancreas compared to WT mice. Moreover, treatment of these mice with the carcinogen azoxymethane led to the formation of pancreatic pre‐cancerous lesions. Recently, Soboh et al. demonstrated the antitumorigenic role of Hpse2 in vivo by promoting macrophages toward a pro‐inflammatory M1 phenotype. They also identified the bromodomain‐containing protein 7, a tumor‐suppressor protein, as an interactor and a shuttle for Hpse2 into the nucleus [76]. Together, these findings reinforce HPSE2 as a tumor suppressor [10, 48] and highlight its endogenous anti‐inflammatory activities [47, 75].

## 5 HPSE1 and HPSE2 in Immune Cells

HPSE1 and HPSE2 have been detected by immunocytochemistry in peripheral blood mononuclear cells isolated from a breast cancer patient, and their levels are higher than in a healthy woman [77]. Concerning Hpse2, its role on immune cells has been recently described. A reduced number of NK cells was observed in the bone marrow and spleen from conditional *Hpse2* ^‐/‐^ mice compared with WT mice. Moreover, macrophages isolated from *Hpse*2^‐/‐^ mice showed a pro‐tumorigenic M2 phenotype, expressed higher levels of the cytokines Il‐6 and Il‐10, favored angiogenesis and promoted tumor growth in vivo compared with macrophages from WT mice [75]. Regarding HPSE1, it is expressed and produced by various cells of the innate immune system, as confirmed by the different mouse models described above, including macrophages [78], neutrophils [79], natural killers (NK) [80], dendritic cells (DCs) [58], mast cells (MCs) [81], eosinophils [82] and platelets [83]. Savion et al. demonstrated that macrophages exhibit heparanase activity, as an exo‐β‐glucuronidase inhibitor decreased HS release in vitro [78]. This was confirmed using macrophages isolated from *Hpse1* ^*−/−*^ mice which exhibited reduced expression of inflammatory markers as well as diminished capacities for migration, phagocytosis and matrigel invasion compared to WT macrophages [66]. The addition of either pro‐ or active Hpse1 to a murine macrophage cell line (J774) or primary murine macrophages led to increased expression of inflammatory markers such as Tnf‐α, Mmp‐9 and Il‐1β. This induction was prevented by inhibitors of the Mapk (mitogen‐activated protein kinase), Pi3k, Nf‐κB and MyD88 signaling pathways. Hpse1‐stimulated macrophages isolated from *Tlr2* ^−/−^ and *Tlr4* ^−/−^ mice showed a decrease in inflammatory marker expression [84]. Overall, these results suggest that Hpse1 acts through Tlr2 and Tlr4, thereby activating Jnk, Erk, p38 and Nf‐κB signaling pathways, leading to increased expression of inflammatory markers as well as enhanced migratory and phagocytic capacities. However, OGT2115, a well‐known synthetic Hpse1 inhibitor, failed to interfere with Hpse1‐mediated inflammatory signaling, underlining the involvement of a non‐catalytic activity of Hpse1 [66]. Furthermore, the addition of recombinant human HPSE1 to PMA‐stimulated U937 cells increased the expression of M1‐type pro‐inflammatory markers such as iNOS, IL‐1β, IL‐6 and TNF‐α. This effect was reversed by SST0001, a chemically modified heparin that inhibits HPSE1 [85]. The influence of Hpse1 on macrophage polarization was further confirmed in chemotherapy‐stimulated macrophages derived from *Hpse1* ^*−/−*^ mice, where a significant shift from anti‐inflammatory macrophages to pro‐inflammatory macrophages was observed [86]. Recently, He et al. demonstrated that OGT2115 limits Hpse1‐driven M2 anti‐inflammatory macrophage polarization and reduces bleomycin‐induced pulmonary fibrosis [87].

In *Hpse1* ^*−/−*^ mice, no morphological or cytokine secretion changes were observed in immature and mature DCs compared to WT mice [58]. However, immature DCs from *Hpse1* ^*−/−*^ mice exhibited increased expression of maturation markers such as Cd80, Mhc class II I‐A/I‐E as well as migration markers Ccr7 and Mmp‐14. Despite this, both in vitro and in vivo experiments revealed a significant decrease in the transmigration of immature and mature DCs from *Hpse1* ^*−/−*^. Poon et al. reported a reduced migration of Langerhans cells and dermal DCs in *Hpse1* ^*−/−*^ mice. Additionally, in a murine model of HDM‐induced allergy, a reduced number of conventional DCs was observed in *Hpse1* ^*−/−*^ mice compared to WT mice [69]. These results collectively underscore the role of Hpse1 in DCs recruitment, migration and transmigration.

HPSE1 expression in eosinophils was demonstrated using RT‐qPCR and immunofluorescence. Co‐localization and direct interaction between HPSE1 and the Major Basic Protein (MBP) were observed in the cytoplasm of mature eosinophils [82]. MBP is synthesized as a pre‐proMBP, containing a secretory leader peptide, a 90‐residue unstructured acidic region linked to the 117‐residue MBP domain that is solely present in mature eosinophils [88]. A significant reduction in HPSE1 activity was observed in MBP‐stimulated eosinophils compared to controls [82], identifying MBP as the first known natural HPSE1‐inhibiting protein with potential protective function in diseases associated with inflammation. The MBP domain, which adopts a folding similar to the C‐type lectin superfamily [89], may be responsible for inhibiting HPSE1. In a murine model of ovalbumin‐induced allergic airway inflammation, a significant decrease in eosinophils number was observed in the airway of *Hpse1* ^*−/−*^ mice, or upon inhibitor treatment, compared to control mice [90].

Matzner et al. showed that heparin antagonized HS degradation by neutrophils, even without prior activation, suggesting that neutrophils constitutively produce and secrete Hpse1 [79]. In Hpa‐Tg mice, administration of the neutrophil‐specific chemokine Mip‐2 decreased neutrophil migration and Mip‐2 staining in the venules, indicating that Hpse1 impairs neutrophil diapedesis by disrupting chemokine sequestration mediated by HS [91]. The important role of HS in neutrophil extravasation was further illustrated in *Ndst‐1* ^*−/−*^ mice, with specific deletion in endothelial cells and leukocytes. Indeed, HS from endothelial cells was particularly required for neutrophil recruitment. Impairment in neutrophil recruitment in these mice correlated with reduced inflammation, transcytosis and Il‐8 binding on endothelial cells [92].

Hpse1 was observed in the nucleus and in clusters at the membrane of immune cells, particularly in NK cells [80]. It was demonstrated that the bromodomain PHD finger transcription factor (Bptf) drives Hpse1 expression in cancer cells, leading to the cleavage of a large portion of HSPGs, which act as co‐ligands of the Natural Cytotoxicity Receptor. Consequently, this cleavage dramatically reduces the NK cytolytic activity against cancer cells [93]. Likewise, NK cells isolated from *Hpse1* ^*−/−*^ mice exhibited decreased cytotoxic activity against a murine lung cancer cell line (EO771.LMB), along with reduced expression of activation markers Cd69 and Nkg2d and lower Mcp‐1 production [94]. These findings provide insight into Hpse1's role in NK cell‐mediated cancer immunosurveillance and its potential as a therapeutic target.

Bashkin et al. showed that mast cells (MCs) can produce and store Hpse1 in their granules and release it to degrade HS on the subendothelial ECM [81]. MCs derived from fetal skin of Hpa‐Tg mice had decreased heparin levels compared to MCs isolated from control mice. Conversely, MCs derived from fetal skin of *Hpse1* ^*−/−*^ mice showed an increase in heparin quantity. Levels of specific proteases such as MC‐Cpa and mMcp‐6 were lower in MCs of Hpa‐Tg mice but higher in those isolated from *Hpse1* ^*−/−*^ mice. Finally, MCs from Hpa‐Tg mice exhibited irregular and smaller granules and released less histamine upon IgE stimulation compared to WT mice [95]. Hpse1 internalization in MCs is controlled by its enzymatic activity, as the latent or inactive form of Hpse1, or the use of an inhibitor, enhanced its uptake, likely due to HSPG increased stability [96].

Wasteson et al. demonstrated that platelets can produce an enzyme that degrades HS [83]. When cultured with endothelial cells and ECM, platelets formed clusters, degraded HS and facilitated lymphoma cell adhesion, suggesting a role of HPSE1 in tumor invasion and metastasis [97]. Platelets from Hpa‐Tg mice had a higher adhesion capacity on endothelial cells than platelets from control mice, contributing to increased thrombotic activity [98]. In patients with sepsis, elevated HPSE1 and activity were correlated with disease‐related mortality [99]. Induction of delayed‐type hypersensitivity (DTH) by oxazolone hapten led to increased ear skin thickness, and strong expression of Hpse1 in keratinocytes and vascular endothelial cells was observed. Skin thickness was increased in Hpa‐Tg mice upon DTH compared to control mice. An important increase in Hpse1 expression and enzymatic activity was observed upon incubation of endothelial cells with Tnf‐α or Ifn‐γ, key mediators of DTH. Finally, treatment with the Hpse1 inhibitor ST1514, a glycol split, non‐anticoagulant heparin, or with anti‐Hpse1 siRNA prevented inflammation, vascular permeability and leukocyte extravasation in the ears [34].

Hpse1 is also produced by cells of the adaptive immune system, such as B lymphocytes [100] and T lymphocytes [101]. In murine B lymphocytes stimulated by LPS, increased Hpse1 enzymatic activity was observed, probably to ease their exit from the bone marrow compartment [100]. Naparsteck et al. showed that rat T cells exhibited an endoglycosidase activity able to release labeled HS upon concanavalin A stimulation [101]. In contrast, T cells activated by anti‐CD3 and anti‐CD28 antibodies exhibited reduced *Hpse1* expression, potentially due to p53 overexpression, which negatively controlled its production [102]. The variability of *Hpse1* expression and activity in T cells seemed to be strongly influenced by the duration of activation. Heparin administration decreased DTH reactions induced by oxazolone, T cell migration, suggesting the involvement of Hpse1 in T cell recruitment and activation [103]. Both active and latent forms of Hpse1 reduce in vitro and in vivo T cell proliferation, activation and cytotoxicity. Indeed, Hpse1 modulates T cell cytokine profiles, with a marked increase in the levels of IL‐4, IL‐6 and IL‐10, and a parallel decrease in IL‐12, TNF‐α and IFN‐γ [104]. This Th2 promoting effect is attributed to a nonenzymatic activity of Hpse1 and could control the inflammatory lesion of murine experimental autoimmune encephalitis [105]. Additionally, Hpse1 increased Th17 differentiation and IL‐17A production [106], further highlighting its multifaceted influence on T lymphocytes, playing different roles depending on the immunological and pathological context.

In a collagen‐induced arthritis (CIA) model using Hpa‐Tg mice, increased numbers of Helios^+^ CD4^+^ and CD8^+^ T lymphocytes were observed in the spleen, thymus or lymph nodes. Th1, Th17 and follicular T cell proportions were also elevated and correlated with heightened inflammation and disease severity [107]. Moreover, a higher proportion of Treg cells was observed in the lymph nodes of CIA in Hpa‐Tg mice without protective action against autoimmunity, justified by a phenotypic shift of Treg cells toward a more inflammatory profile [107]. In the T cell‐mediated inflammation model, it was found that deletion of *Hpse1* in the CD4^+^ T cell population led to a decrease in inflammation and recruitment of macrophages, CD4^+^ and CD8^+^ T cells [108]. Altogether, these results highlight a crucial role of Hpse1 in T cell‐mediated inflammation.

ProHpse1 also binds to Hspgs to facilitate T cell rolling and adhesion on major components like Cd44 and fibronectin. Additionally, Hpse1 increases the expression of Sdf‐1α promoting T cell recruitment [109]. In chimeric antigen receptor T (CAR‐T) cells, overexpression of *Hpse1* enhanced tumor cell elimination without compromising their viability or function. In vivo, these engineered CAR‐T cells showed increased infiltration in tumors, underscoring the ability of Hpse1 to degrade the ECM, improve immune cell infiltration around tumors and promote antitumor immunity [102].

Overall, these studies demonstrated the implication of Hpse1 in immune cells homeostasis, influencing their activation, polarization, migration, proliferation and differentiation.

## 6 Roles of HPSE1 on Skin Pathologies

Given its essential protective functions, the skin is permanently exposed to pathogens, allergens, UV radiation and pollution. This necessitates a complex and finely tuned crosstalk between structural cells such as keratinocytes and fibroblasts, and the whole immune cells described above [110]. Consequently, the skin is a relevant organ for investigating the role of both heparanases in pathophysiological conditions.

The first identification of pro‐ and active forms of HPSE1 in human skin was reported in 2001, in the plantar *stratum corneum*, in reconstructed epidermis and in normal human skin [111]. HPSE1 was detected from the *stratum granulosum* to the upper layers of the *stratum corneum* and in Langerhans cells [111]. During the calcium‐induced differentiation of normal human keratinocytes (NHEK), a translocation of HPSE1 from the cytoplasm to the nucleus was observed, leading to the degradation of nuclear HS and altered expression of the differentiation markers p27 and involucrin [112]. Inhibition of HPSE1 increased HS deposition at the dermal–epidermal junction (DEJ), enhanced expression of the differentiation markers loricrin and filaggrin, and elevated the number of Ki67‐positive basal keratinocytes [113, 114]. In models of shaving or chemotherapy‐induced alopecia in Hpa‐Tg mice, keratinocyte‐derived Hpse1 was shown to promote hair growth by stimulating vascularization, maturation and keratinocyte migration [54].

Hpse1 is also expressed in the granulation tissue in mouse and rat models of wound healing. In vitro wound healing assays demonstrated that recombinant HPSE1 enhanced HaCaT keratinocyte migration. Furthermore, topical application of recombinant HPSE1 or the HPSE1‐activating antibody 6F8 accelerated wound healing. This has been confirmed in Hpa‐Tg mice, which exhibited enhanced wound epithelialization, epidermal thickening and blood vessel maturation [115, 116]. In a murine model of repeated skin injury, increased *Hpse1* expression was correlated with reduced levels of HS and HS‐bound Fgf‐2 and Vegf‐a at the DEJ. Topical application of a Hpse1 inhibitor reduced HS degradation, blood and lymphatic vessel size and density [117]. Notably, HS levels are higher in the skin of older rats compared to younger ones, correlating with increased skin thickness and decreased *Hpse1* expression, underlining a role of Hpse1 in skin aging and homeostasis [118].

*HPSE1* expression is dysregulated during skin inflammation. In UVB‐irradiated skin and UVB‐irradiated NHEK, increased HPSE1 expression is associated with diminished levels of HS at the DEJ and in the epidermis, thus reducing the binding of VEGF‐A, FGF‐2 and FGF‐7. In this context, the dual inhibitor of HPSE1/MMP‐9 inhibitor, BIPBIPU, decreased damage to the basement membranes at the DEJ, as well as epidermal proliferation, differentiation, water retention and transepidermal water loss [119]. In *solar lentigo*, characterized by a harmless patch of darkened skin, high expression of HPSE1 and an increase in the size and number of blood vessels are associated with decreased levels of HS at the DEJ compared to non‐pigmented skin. In pigmented reconstructed skin models, HPSE1 inhibition by BIPBIPU restored HS levels and reduced melanogenesis. These findings suggest that HS degradation by HPSE1 enhances growth factors transfer from the dermis to melanocytes, thereby promoting melanogenesis [120].

HPSE1 expression is also increased in keratinocytes from human psoriatic skin lesions as demonstrated by RT‐qPCR [106, 121, 122] and by immunohistochemistry [119]. Zhu et al. reported decreased *HPSE1* expression in lesional tissues of psoriasis patients treated with an anti‐IL‐17A antibody, whereas IL‐17A of HaCaT increased HPSE1 production [106]. In murine models of psoriasis induced by 12‐O‐tetradecanoyl phorbol 12‐myristate 13‐acetate (TPA) or by topical application of imiquimod (IMQ), Hpse1 expression was also increased [106, 121]. In TPA‐treated Hpa‐Tg mice, increased epidermal thickening, hypervascularity, increased Stat3 phosphorylation and greater macrophage infiltration were observed compared to WT mice [121]. In the IMQ model, co‐treatment with OGT2115 and anti‐Il‐17A antibody decreased Th17 cell numbers [106]. These results demonstrate the contribution of Il‐17A to *Hpse1* upregulation and implicate HPSE1 in psoriasis pathogenesis through macrophage and Th17 cell recruitment and hypervascularization of psoriatic lesions.

Regarding HPSE2, Wagner et al., observed an increased mRNA and protein expression in psoriatic lesions compared to non‐lesioned skin [122]. In contrast, Samaka et al. reported a total absence of HPSE2 by immunohistochemistry in psoriatic skin lesion [123], A discrepancy likely due to the use of different primary antibodies.

Altogether, these findings underscore the important role of HPSE1 in immune responses and in skin pathophysiology. However, the function of HPSE2 remains unclear. Many questions persist regarding the physiological relationship between these two proteins. Has this relationship existed across evolution? Have these genes co‐evolved across species? How conserved are their structures and functions, particularly in species used as preclinical models? Finally, is it possible to demonstrate or predict how HPSE1 and HPSE2 could interact? To address these questions, we employed phylogenetic analyses and AlphaFold (AF3) molecular predictions [124].

## 7 Phylogeny Analysis of *HPSE1* and *HPSE2* Genes

**FIGURE 2:**
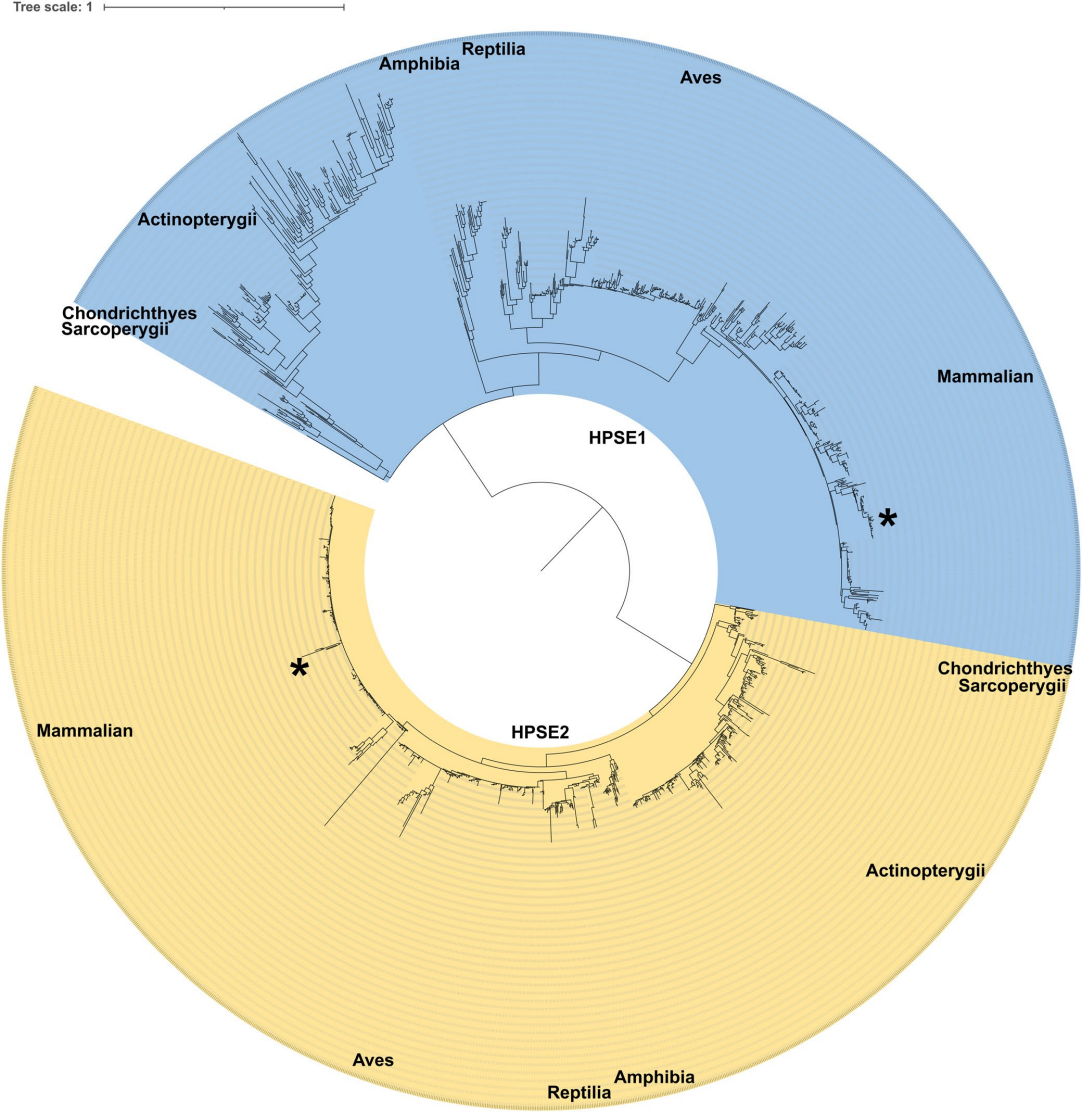
Phylogenetic analysis of HPSE1 and HPSE2 sequences showing the early divergence between the two heparanases. Phylogenetic tree of aligned protein sequences of Hpse1 and Hpse2c orthologs. Protein sequences were aligned using MAFFT [126], clustered with CD‐HIT and curated using BMGE [127]. The tree was generated with IQ‐TREE [128] and displayed with iTOL [129]. The two groups are colored in cyan and yellow/orange with the position of human Hpse1 and Hpse2c indicated by an asterisk; the main organism classes are labeled.

HPSE2 can be classified as a pseudoenzyme in sharing a similar fold with canonical HPSE1 but devoid of catalytic activity [125]. To clarify the evolution history of *HPSE2*, we retrieved 928 and 1134 sequences of *HPSE1* and *HPSE2*, respectively, from the NCBI GenBank database. Given the overall homology between *HPSE1* and *HPSE2*, we applied a ~50% sequence identity cutoff to reduce sequence heterogeneity. After clustering to minimize sequence redundancy, we constructed a phylogenetic tree based on a multiple sequence alignment of 871 HPSE1 and 1030 HPSE2 sequences to trace lineage and identify a possible common ancestor of all *Hpse1* and *Hpse2* sequences (Figure 2). The earliest diverging outgroup at the base of the tree for *Hpse1* and *Hpse2* homologs unambiguously indicates that these two genes originated from two different families with no direct information about the origin of the “loss‐of‐function” event(s) or the lineage separation. Instead, it is likely that HPSE2 acquired a new, non‐catalytic function following gene duplication from a genuine HPSE1 ancestor, as proposed for most pseudoenzymes [125]. The presence of the *Hpse1* and *Hpse2* genes in major metazoan lineages suggests that their functions are likely essential. Interestingly, current data indicate that functional *Hpse1* and *Hpse2* genes are absent in insects and nematodes [130], even though HSPGs appear to perform similar functions in these organisms as in mammals [131, 132]. Based on conserved sequences and the phylogenetic analysis, the cladogram shows that *Hpse2* has evolved into two main subgroups and several clades. Human *HPSE2* gene is located in the largest subgroup, which is divided into two principal clades. These findings suggest that Hspgs and Hpse1 are present across Metazoa including Mammalia, Aves, Amphibia, Reptilia, and Actinopterygii. Hpse2, which is absent in Ecdysozoa and Lophotrochozoa, has likely been retained to regulate specific processes through catalytically independent mechanisms.

**FIGURE 3:**
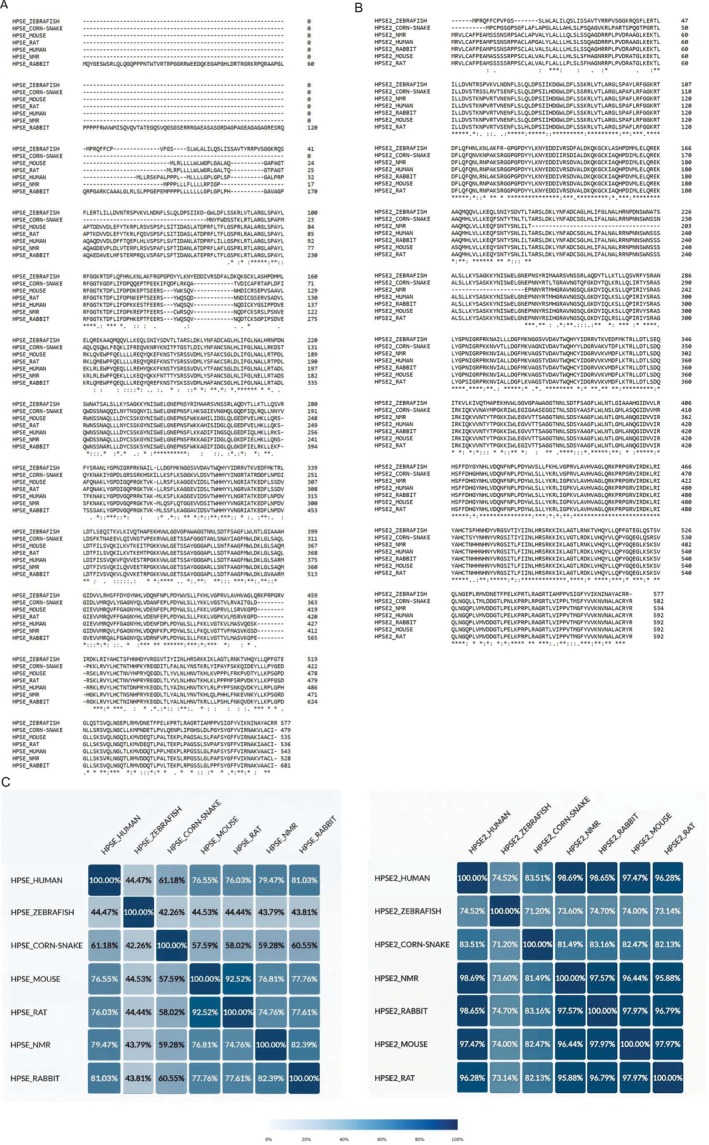
Multiple sequence alignment of (A) Hpse1 and (B) Hpse2 performed using CLUSTAL O (1.2.4) multiple sequence alignment (https://www.uniprot.org/align) with the default parameters to identify conserved regions. All the protein sequences were obtained from the UniProt database. (C) Percentage of sequence identity between HPSE1 or HPSE2 from *Homo sapiens* (Human), *Heterocephalus glaber* (Naked Mole‐Rat, NMR), *Oryctolagus cuniculus* (Rabbit), *Mus musculus* (Mouse), *Rattus norvegicus* (Rat), *Pantherophis guttatus* (Corn snake) and *Danio rerio* (Zebrafish). Identical residues are marked with an asterisk (*), strongly similar residues with a colon (:) and weakly similar residues with a period (.). HPSE2 in (B) and (C) refers to the longest human isoform HPSE2c.

To complement this analysis, we performed primary sequence alignments to assess global sequence identity of Hpse1 and Hpse2 across multiple species (Figure 3A,B) *Homo sapiens* (human), *Heterocephalus glaber* (naked mole‐rat), *Oryctolagus cuniculus* (rabbit), *Mus musculus* (mouse), *Rattus norvegicus* (rat), *Danio rerio* (zebrafish) and *Pantherophis guttatus* (Corn snake), a phylogenetically distant species (Figure 2). The sequence alignment of Hpse2 across these selected species revealed a 91% identity, indicating a relatively high degree of conservation. In contrast, Hpse1 showed a lower identity of approximately 70%, suggesting that Hpse2 is more conserved across species than Hpse1 (Figure 3C).

## 8 Analysis of HPSE1 and HPSE2 by Molecular Modeling

### 8.1 Predicted Structures of the Four Human HPSE2 Isoforms

In contrast to the mature, active form of HPSE1, which consists of a proteolytically processed non‐covalent heterodimer composed of an 8 kDa N‐terminal and a 50 kDa C‐terminal subunits, the four HPSE2 isoforms consist of a single, non‐processed polypeptide chain (Figure 1B). The predicted AF3 model of human HPSE2c (Gly41‐Arg592) adopts a similar overall architecture to HPSE1, consisting of a (β/α)_8_ domain flanked by a small β‐sandwich domain, as reported in the crystal structure of human HPSE1 [133] (Figure 1B, with an rmsd value of 1.1 Å for 480 Cα atoms). While HPSE1 contains 6 putative N‐glycosylation sites (all located on the 50 kDa subunit, with 5 visible in the crystal structure), HPSE2c has only 4 such sites, of which three (Asn237, Asn254, and Asn275) are conserved with HPSE1. The disulfide bonds pattern is also conserved between the two proteins. The AF3 models of the three other HPSE2 isoforms, namely HPSE2a, HPSE2b and HPSE2‐2, display similar global architectures, with notable surface differences in the (β/α)_8_ domain resulting from the presence or absence of specific sequence segments (Figure 1B). In HPSE2b, for example, the region corresponding to Glu221 in HPSE1, which lies within the vestigial substrate‐binding cleft in other HPSE2 isoforms is displaced to the protein surface due to the insertion of a spliced segment spanning Asp150‐Thr202. Such structural rearrangements in HPSE2b and HPSE2‐2 may influence potential interactions or partnerships with HPSE1 or other binders.

A structural overlay of HPSE1 and HPSE2c highlights two regions of major conformational variability (Figure 1C). First, and as expected, the region spanning residues Arg129 to Asn195 in HPSE2c, corresponding to the 6 kDa linker peptide in the structure of proHPSE1, which is cleaved by CTSL to generate mature HPSE1, features two inserted helices that protrude from the (β/α)_8_ domain. Second, a loop insertion of 9 residues (Arg466‐Arg475), in the β‐sandwich domain of HPSE2c contains 5 basic residues conserved among the four isoforms. Structural overlays of the five HPSE2c predicted models show conformational flexibility in both surface regions (data not shown).

The substrate binding cleft characteristic of HPSE1, which is lined with basic residues, is absent in HPSE2c. This is preliminary due to the presence of the large α‐helix (Asp171‐Ser194) that forms a lid over the vestigial active site. This topology is reminiscent to the corresponding large helical Pro134‐Gln157 domain in proHPSE1 that blocks access to the bulky HS substrates [134]. Moreover, the catalytic machinery in HPSE2c is non‐conserved and vestigial: residues Gly381 and Glu262 correspond to the nucleophile Glu343 and the acid–base Glu225 in HPSE1. An overlay of the HPSE2c model with the HPSE1 crystal structure bound to a heparin tetrasaccharide (PDB ID: 5E9C) reveals steric clashes between the C‐terminal region of the helix lid (Leu189‐Gln192) and the substrate (Figure 1C). Of the eight basic residues lining the HPSE1 binding cleft, only three (Arg272, Lys273 and Arg303) are conserved in HPSE2c. This results in a distinct surface electrostatic potential distribution between the two proteins (Figure 1A,B). While HPSE1 exhibits a prominent positively charged patch around the binding cleft and at the top of the β‐sandwich domain, HPSE2c displays a similar positive charge cluster within the β‐sandwich domain, extending into the (β/α)_8_ domain. This distribution is consistent with the higher heparin/HS‐binding affinity observed for HPSE2c compared to HPSE1 [43]. In the HPSE2c model, three heparin‐binding consensus motifs, i.e., Q_465_RKPRP_470_, N_486_HHNHN_491_ and L_504_HRSRKKIKL_513,_ are defined. These correspond to Cardin‐Weintraub motifs, defined by the sequences XBBXBX and XBBBXXBX, where B is a basic amino acid such as Arg, Lys, rarely His and X is a neutral or hydrophobic residue. Notably, these three motifs are clustered within surface loops of the β‐sandwich domain (Figure 1B).

### 8.2 Predicted Structure of the Human HPSE1‐HPSE2c Complex

**FIGURE 4:**
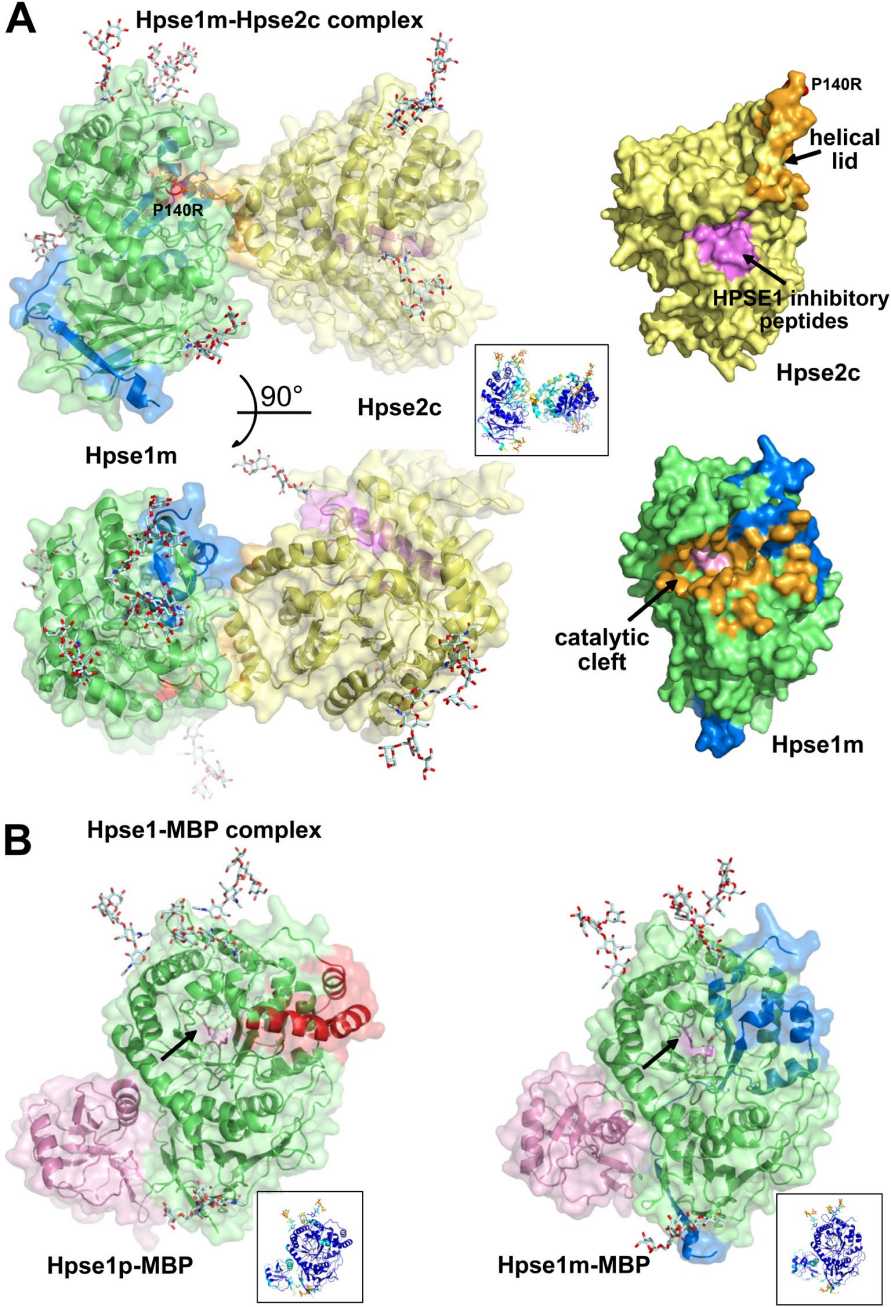
Overall views of the AF3 predicted models of the HPSE1‐HPSE2c and HPSE1‐MBP complexes. (A) (Left) The two HPSE1 and HPSE2c molecules from the top‐ranked predictions of the human HPSE1‐HPSE2c complex are shown in two orientations rotated by 90° with a transparent surface and colored as in Figure 1 (colored by prediction confidence pLDDT score as inset). (Right) Overall views, oriented as in Figure 1, of the buried interfaces colored in orange at the molecular surface of HPSE2c (top) and HPSE1 (bottom) in the predicted complexes. Steric interactions of the helical lid of HPSE2c within the HPSE1 binding cleft with the two catalytic residues colored in pink are evident. The location of the human HPSE2c Pro140Arg variant at the tip of the helical lid, associated with the UFS rare disease, is highlighted in red and labeled; those of the two HPSE2c peptides reported to inhibit HPSE1 are highlighted in pink. (B) Overall views of the top‐ranked AF3 predictions for (left) pro (linker region colored in red) and (right) mature HPSE1 bound to the MBP C‐type lectin domain shown with a transparent surface with the HPSE1 molecule oriented and colored as in A. The catalytic cleft is indicated by an arrow with the two catalytic residues colored in pink. A similar binding mode highlighting a shape complementarity at the interface of the two proteins is observed.

Given the physical interaction between HPSE1 and HPSE2c observed by co‐immunoprecipitation [43] and the mapping of HPSE1‐inhibitory HPSE2c peptides [44], we used AF3 [124] to further investigate the architecture of the HPSE1‐HPSE2c complex. Predictions were performed using pro and mature forms of HPSE1, as well as the stable P6 [135] and the constitutively active GS3 variants [136]. To our knowledge, no experimental data currently evaluate the capacity of the three other non‐secreted HPSE2 isoforms to interact with HPSE1, HS and/or HSPG that raise questions about the biological relevance of such complex. For the HPSE1‐HPSE2c complex, the binding interface spans ~1100 Å^2^, with the 50 kDa subunit of HPSE1 contributing slightly more (578 Å^2^) than the 8 kDa subunit (519 Å^2^), consistent with an expected “moderate” binding affinity. In this model, the tip of the large helical lid of HPSE2c (residues Gly136‐Glu148, which includes three tyrosine residues) inserts into the HPSE1 binding cleft. This suggests a potential inhibitory mechanism, where HPSE2c sterically hinders access to the HPSE1 active site. Notably, the active site of HPSE1 and the vestigial active site of HPSE2c face in opposite directions (Figure 4A), as previously proposed [43] (Figure 4A). At the interface, residues in the tip of HPSE2c's helical lid, along with those in the N‐cap of the α‐helix (Pro170‐Gln177), interact with multiple residues surrounding HPSE1's substrate‐binding cleft, effectively blocking substrate access. A comparison between the bound and unbound HPSE2c models reveals significant conformational flexibility in the lid tip (Arg129‐Lys145), which undergoes a ~19 Å pivot to engage with HPSE1 upon binding. Interestingly, the HPSE2c peptides previously shown to inhibit HPSE1 activity [44], particularly, the two most effective, spanning residues 381–410 and 401–430, are not located near the predicted binding interface in the complex model (Figure 4A), a discrepancy that warrants further investigation. A similar binding interface is observed in AF3 predictions using the stable P6 variant or the active GS3 HPSE1 variant, although the orientation of HPSE2c differs slightly (Table 1) [135, 136]. When the proHPSE1 sequence, which includes a large helical lid above the active site cleft (accession code 5la4), is used for modeling, the position of HPSE2c shifts, but the overall orientation of the two proteins remains comparable.

**TABLE 1:** The AlphaFold scores for the proteins and complexes studied.

Protein/Complex	pTM	ipTM	pLDDT
HPSE2a	0.78	0.67	73.57
HPSE2b	0.73	0.65	66.91
HPSE2c	0.87	0.84	82.65
HPSE2‐2	0.81	0.76	73.84
HPSE1P6‐HPSE2c	0.53	0.41	80.05
pHPSE1‐HPSE2c	0.56	0.41	84.81
HPSE1GS3‐HPSE2c	0.54	0.4	80.11
mHPSE1‐HPSE2c	0.55	0.44	80.89
pHPSE1‐MBP	0.85	0.72	88.29
mHPSE1‐MBP	0.82	0.81	88.96
HPSE1GS3‐MBP	0.82	0.67	90.20
HPSE1P6‐MBP	0.83	0.82	89.22

*Note:* pHPSE1: proHPSE1; mHPSE1: mature HPSE1; HPSE1 P6 and HPSE1 GS3 are described by Whitefield et al. [135] and Nardella et al. [136] respectively. https://www.ebi.ac.uk/training/online/courses/alphafold/inputs‐and‐outputs/evaluating‐alphafolds‐predicted‐structures‐using‐confidence‐scores/confidence‐scores‐in‐alphafold‐multimer/.

Abbreviations: ipTM, interface predicted template modeling; pLDDT, predicted local distance difference test; pTM, predicted template modeling.

Although most pathogenic HPSE2 mutations associated with UFS are stop‐gain, splice site or deletion variants, two missense mutations Asn532Ile [137] and Pro140Arg [138] have been reported. Mapping these mutations onto the HPSE1‐HPSE2c complex reveals that only the Pro140Arg mutation, located at the tip of the helical lid and buried at the binding interface, is likely to disrupt complex formation (Figure 4A). Finally, we investigated the mode of binding of HPSE1 in complex with the MBP domain. High‐confidence AF3 models show that MBP anchored to HPSE1's surface primarily through helix α1, burying ~1000 Å^2^ of surface area (576 Å^2^ on the 50 kDa subunit and 423 Å^2^ on the 8 kDa subunit), and positioned away from the active site pocket (Figure 4B). A similar MBP binding mode is observed in models using both the GS3 variant or the proHPSE1 (Figure 4B), suggesting a possible allosteric inhibitory mechanism that could induce dynamic structural changes in HPSE1, a hypothesis that warrants further investigation.

## 9 Concluding Remarks and Future Perspectives

In humans and murine preclinical models, HPSE1 functions are frequently, if not consistently, linked to the biology of HSPGs at the post‐synthetic level [45, 139]. Inhibitors such as heparinoids or HS‐derived molecules, which act either directly or indirectly on HPSE1, and possibly HPSE2, have demonstrated numerous beneficial effects [140]. In vitro and in vivo studies have shown that targeting HPSE1 activity in various cancer cells inhibits cell migration and invasion, while promoting autophagy. However, clinical trials with HPSE1 inhibitors have yielded encouraging but not yet successful results. This may indicate that the inhibitors are not being delivered at the appropriate site of HPSE1 activity, outside or inside the cells, in lysosomes or nuclei, or that they are not administered within the optimal therapeutic window. Moreover, according to the importance of the C‐terminal domain in non‐enzymatic activity of HPSE1 [141], or the pro‐tumorigenic activities of the T5 variant of HPSE1 [15], it is also possible that HPSE1 enzymatic activity is not solely responsible for the observed pathophysiological effects in vivo. Despite their seemingly opposite expression profiles and roles in cancer and inflammation, HPSE1 and HPSE2c share a key pathophysiological feature: the significance of their subcellular localization. The two heparanases can be secreted, anchored at the membrane, or localized in the nucleus. In these compartments, they may interact with histones, modulate chromatin accessibility and function as transcriptional regulators [47, 142, 143, 144, 145]. A better understanding of their intracellular trafficking, localization and functions, for example the characterization of HPSE1 activity in lysosomes in normal vs. cancer cells, along with the identification of their interacting partners, is essential for designing novel targeted therapeutics.

Interestingly, across species expressing both heparanases, the Hpse1 sequences appear to be more variable than those of Hpse2, suggesting that Hpse2 may possess more conserved functional features, a hypothesis that remains to be fully investigated. This also raises evolutionary questions. For instance, while HSPGs metabolism is critical for normal development and function in invertebrates such as *Drosophila melanogaster* and *Caenorhabditis elegans* , neither HPSE1 nor HPSE2 has been clearly identified in these organisms. Although proteins annotated as “heparanase” exist in *D. melanogaster* and the annelid *Capitella teleta*, their primary sequences are highly divergent from those of HPSE1 (data not shown). This suggests the existence of functional surrogates, such as sulfatases [131, 132] or proteases [146, 147], which may fulfill similar roles. Proteases could mediate the shedding of HSPGs from the cell surface, while sulfatases may modulate the interaction between HS chains and their binding partners (e.g., morphogens, growth factors and cytokines). Increasing evidence supports the notion of a complex interplay between sulfatases, endoglycosidases and proteases. These three classes of post‐synthetic HSPG‐modifying enzymes are all implicated in vital biological processes such as embryonic development, wound healing, inflammation and cancer [148]. HPSE1 itself has been shown to promote HSPGs shedding [149]. Activation of the endosulfatase Sulf‐2 depends on the removal of its glycosaminoglycan chain by hyaluronidase family enzymes [150], and possibly by sheddases. Additionally, heparin, which is a potent HPSE1 inhibitor, is also a preferred substrate of Sulfs [151]. Together, these observations open up exciting opportunities to explore synergies between HPSE1, sulfatases and proteolytic shedding proteases.

What, then, is the fundamental role of HPSE1? Several single nucleotide polymorphisms in *HPSE1* introns have been associated with disease [152, 153], yet no coding mutations in humans have been linked to disease‐causing HPSE1 protein variants [3]. Furthermore, *Hpse1* ^−/−^ mice are viable and display only mild phenotypes [55]. Most in vivo studies on Hpse1 and Hspgs have relied on transgenic overexpression models (Hpa‐Tg) [54] or constitutive *Hpse1* ^−/−^ mice [55]. However, given HPSE1's low physiological expression across most tissues, its role may be overestimated in some contexts. Conditional *Hpse1* ^−/−^ models have revealed functional discrepancies, suggesting tissue‐specific roles that may have been overlooked in earlier studies [69]. These models will be particularly valuable for studying cell‐specific functions, for example, in skin diseases such as psoriasis. In contrast, data on HPSE2 remain limited, but conditional knockout models have already demonstrated and validated its tumor‐suppressive and anti‐inflammatory functions [47, 75]. Future development of mouse models with inducible, tissue‐specific expression of Hpse1 and Hpse2 will be crucial to precisely define their physiological roles (Baranger, Bourne, Morel, personal communications). In conclusion, the study of HPSE1 and HPSE2 in pathophysiology remains a significant challenge, but one that must be met in order to fully understand their biological roles and to develop more effective therapeutic strategies.

## Author Contributions

E.V., C.D., H.M. and R.H. drafted the manuscript. E.V. and Y.B. created the pictures. All the authors discussed, wrote and approved the manuscript. F.M., Y.B. and K.B. supervised the writing of the manuscript.

## Conflicts of Interest

The authors declare no conflicts of interest.

## Data Availability

Data sharing not applicable to this article as no datasets were generated or analyzed during the current study.
